# The Mediating Effect of Central Obesity on the Association between Dietary Quality, Dietary Inflammation Level and Low-Grade Inflammation-Related Serum Inflammatory Markers in Adults

**DOI:** 10.3390/ijerph20053781

**Published:** 2023-02-21

**Authors:** Shuai Zhang, Xuebin Yang, Limei E, Xiaofei Zhang, Hongru Chen, Xiubo Jiang

**Affiliations:** Department of Epidemiology and Health Statistics, School of Public Health, Qingdao University, Qingdao 266021, China

**Keywords:** dietary score, diet quality, obesity, inflammatory marker, CRP

## Abstract

To date, few studies have explored the role of central obesity on the association between diet quality, measured by the health eating index (HEI), inflammatory eating index (DII), and low-grade inflammation-related serum inflammatory markers. In this paper, we use the data from the 2015–2018 National Health and Nutrition Examination Survey (NHANES) to explore this. Dietary intakes were measured during two 24-h dietary recall interviews and using USDA Food Pattern Equivalence Database (FPED) dietary data. Serum inflammatory markers were obtained from NHANES Laboratory Data. Generalized structural equation models (GSEMs) were used to explore the mediating relationship. Central obesity plays a significant mediating role in the association between HEI-2015 and high-sensitivity C-reactive protein (hs-CRP), mediating 26.87% of the associations between the two; it also mediates 15.24% of the associations between DII and hs-CRP. Central obesity plays a mediating role in 13.98% of the associations between HEI-2015 and white blood cells (WBC); it also mediates 10.83% of the associations between DII and WBC. Our study suggests that central obesity plays a mediating role in the association of dietary quality with low-grade inflammation-related serum inflammatory markers (hs-CRP and WBC).

## 1. Introduction

Growing evidence shows that low levels of chronic systemic inflammation are associated with a number of chronic diseases, including cardiovascular disease, cancer, chronic kidney disease and neurodevelopmental disorders [1,2,3]. Dietary nutrition is a key variable affecting chronic inflammation, mainly because daily food intake is a good indicator of inflammatory potential [4].

Recent studies have linked different types of food to chronic inflammation. A previous study has shown that when added sugars are consumed, fat cells release pro-inflammatory cytokines that trigger inflammation [5]. Recent studies have also found an inverse association between increased vegetable and fruit intake and serum CRP levels [6,7]. The results of a cross-sectional study in India showed that a 1% reduction in dietary saturated fatty acid (SFA) intake was associated with a 0.14 g/L reduction in plasma hs-CRP, after adjusting for relevant variables [8]. Although the relationship between individual foods or individual nutrients and chronic inflammation has been discussed, in recent years, there has been a growing recognition that different combinations of food components may interact in complex ways that are better explained by dietary patterns.

Dietary intake can modulate cancer and is a promising means of reducing the risk of chronic diseases and metabolic dysfunction [9,10,11,12]. Healthy eating index (HEI) score is a measure of dietary quality, which represents the degree to which the Dietary Guidelines for Americans (DGA) are followed [13,14]. Dietary inflammatory index (DII) score is a measure of dietary inflammatory potential based on the overall inflammatory characteristics of dietary components [15,16]. Several studies [9,12,16] have shown that both HEI and DII are associated with inflammatory markers.

Obesity is described as a chronic low-grade inflammatory state, and visceral fat is known to secrete a number of inflammatory markers [17,18]. The increased secretion of adipokine in people with obesity may lead to chronic low-grade inflammation and oxidative stress, which may induce the development of chronic diseases [19]. Studies [20,21,22,23,24] have shown that DII and HEI score were associated with central obesity. However, the underlying mechanism between these diet scores and chronic systemic inflammation remains unclear. Therefore, it is necessary to explore the pathways and intrinsic associations between dietary scores and inflammation. Thus, changes of body weight under different dietary conditions may lead to changes in inflammatory markers in the body.

Taken together, the above evidence suggests that central obesity may be a causal chain between dietary scores and chronic inflammation. However, to the best of our knowledge, no study to date has investigated whether central obesity mediates the relationship between diet score and inflammatory markers. Therefore, the aim of this study was to explore the relationship between DII, HEI and the level of inflammatory markers, and to further explore whether this relationship is mediated by obesity.

## 2. Materials and Methods

### 2.1. Data Source and Study Sample

The data of this study were obtained from National Health and Nutrition Examination Survey (NHANE) multi-stage large sample database. NHANES is a cross-sectional study conducted by the National Center for Health Statistics (NCHS) and the Centers for Disease Control and Prevention (CDC) that provides data from a nationally representative survey of the health and nutrition status of the non-institutional United States (U.S.) population. It follows complex multi-stage sampling design, investigation including face to face interviews at home (population, social economy, diet, and health related issues), in the center of the flow check health checks (medical and physiological measurement) and laboratory test (exposure biomarkers and end). One cycle in NHANES includes data collected by two years.

**Figure 1:**
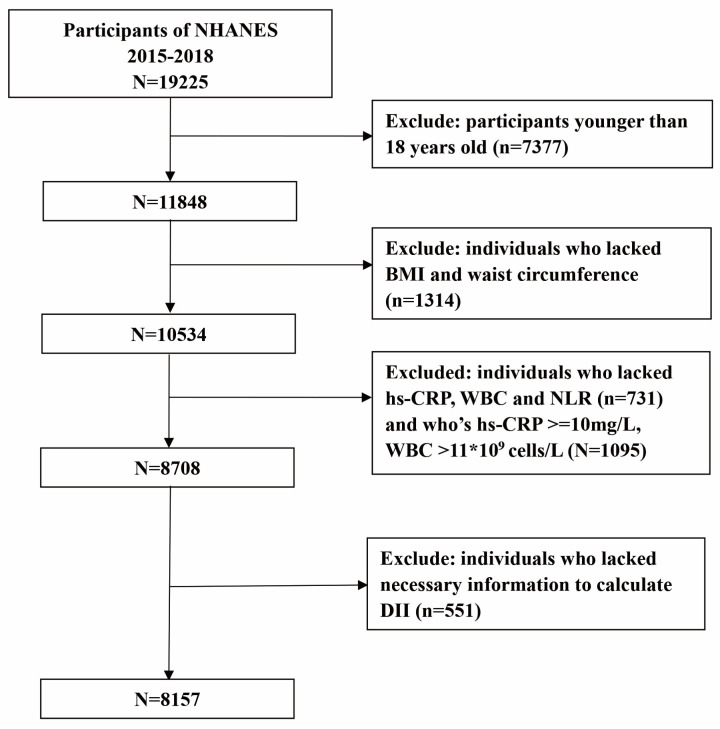
Flow chart of the screening process for the selection of eligible participants.

Data from NHANES, from the years 2015–2016 and 2017–2018, were selected for this study, which included a total of 19,225 participants. 7377 participants were under the age of 18 years old and 1314 lacked data for BMI and waist circumference. 731 participants lacked the data for high-sensitivity C-reactive protein (hs-CRP), white blood cells (WBC), and neutrophil to lymphocyte ratio (NLR). 1095 participants with abnormal values that could not reflect the state of low-grade inflammation well (hs-CRP ≥ 10 mg/L [25] and WBC >11 × 10^9^ cells/L [26]) were excluded. Thus, a total of 8157 participants were enrolled in our study (Figure 1).

### 2.2. Central Obesity

Central obesity was defined by waist circumference, which was defined as waist circumference ≥ 102 cm in men and ≥ 88 cm in women. Although BMI is a common indicator of obesity in general, it does not reflect differences in the distribution of body fat between individuals [27].

### 2.3. Dietary Score

In this study, two different types of dietary scores were selected. We used the healthy eating index (HEI) score to represent dietary quality in participants. The dietary inflammatory index (DII) score was used to represent inflammatory dietary index.

#### 2.3.1. Healthy Eating Index (HEI)

We use the HEI score that was designed and recommended by the United States Department of Agriculture (USDA) to measure an individual’s adherence to the dietary guidelines for Americans (DGA) [28]. The HEI-2015 score is the latest version diet index based on the HEI. The maximum score of HEI-2015 is 100. This index consists of 13 components, which can be divided into 9 adequacy components (total vegetables, greens and beans, total fruits, whole fruits, whole grains, dairy, total protein foods, seafood, plant proteins, and fatty acids) and 4 moderation components (sodium, refined grains, saturated fats, and added sugars). The more adequacy components are consumed, the higher the score, while the fewer moderation components are consumed, the higher the score.

NHANES individual food questionnaire data and Food Patterns Equivalents Database (FPED) dietary data were used to estimate food supply to determine the HEI-2015 score. Each food was classified according to the USDA food code. Finally, the recommended SAS code was used to calculate the HEI-2015 score [29].

#### 2.3.2. Dietary Inflammatory Index (DII)

**Figure 2:**
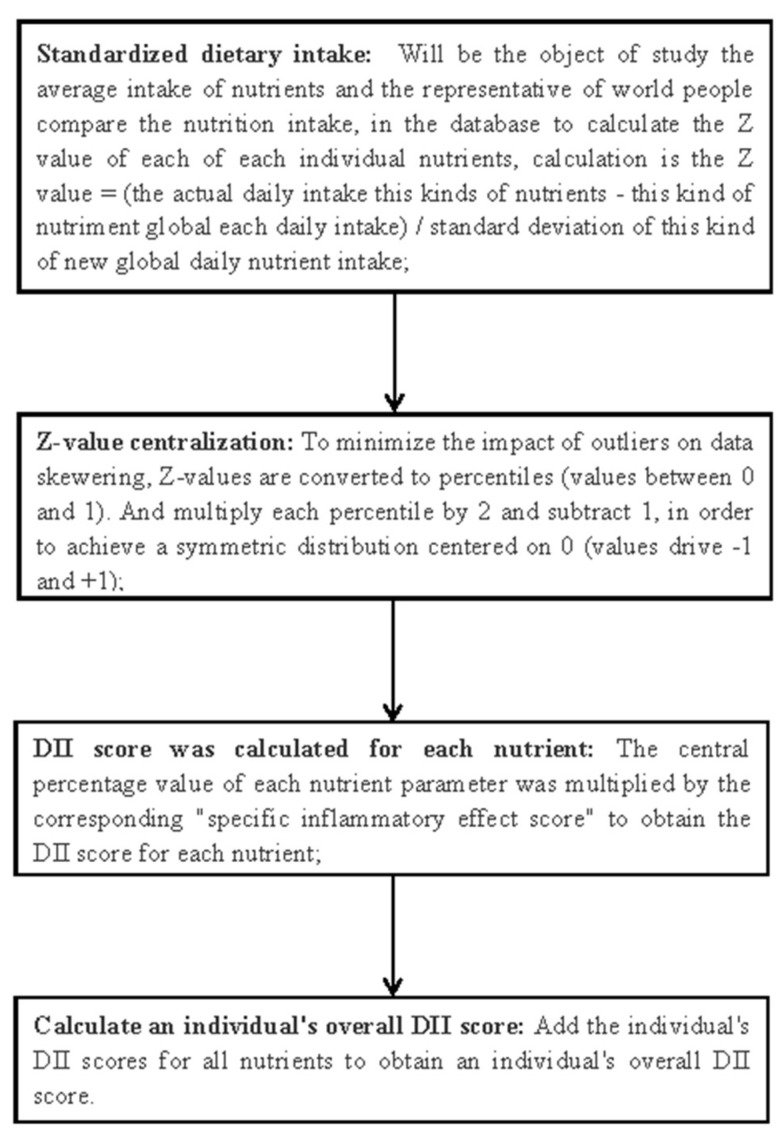
The calculation flow of DII.

DII score is an indicator of the inflammatory potential of foods and can be used in all populations where dietary data can be collected. DII calculations involved 45 dietary parameters, including a variety of macro and micronutrients, flavonoids, flavorings, and other bioactive compounds, each of which correlated with inflammatory effect scores. Then, DII scores were calculated as a standardization of the world database, which contains the mean and standard deviation of food intake parameters from 11 countries around the world. In NHANES, 45 dietary parameters and 28 inflammatory parameters (carbohydrate, protein, cholesterol, iron, zinc, magnesium, selenium, fiber, fat, monounsaturated fatty acids, caffeine, n-3 polyunsaturated fatty acid, n-6 polyunsaturated fatty acids, polyunsaturated fatty acids, saturated fatty acid, alcohol, vitamin A, vitamin B1, vitamin B2, vitamin B6, vitamin B12, vitamin B6, vitamin B12, beta-carotene, vitamin C, vitamin D, vitamin E, folic acid, energy) can be used for DII score calculation. Previous studies have shown no change in DII’s ability to predict inflammation when the available food parameters are reduced, compared with a complete study with 45 parameters [15,30]. DII scores > 0 indicates that the individual’s diet has a pro-inflammatory effect; DII scores < 0 indicates that the diet has anti-inflammatory effects. The specific DII calculation process is shown in Figure 2.

### 2.4. Serum Inflammatory Marker

Serum inflammatory markers were obtained from NHANES laboratory data. Three different inflammatory markers were selected, including hs-CRP, WBC, and NLR.

CRP is a sensitive marker of systemic inflammation, tissue damage and infection in clinical practice [31]. It is one of the sensitive but non-specific inflammatory indicators. Compared with simply calculated CRP, hs-CRP can reflect the current level of cardiovascular disease risk in individuals without inflammatory conditions. In NHANES, CRP was quantified by latex-enhanced turbidimetry. Because laboratories, instruments, and methods varied between the two periods we explored, weighted Deming regression provided by NHANES was used to compare the two [32]. The equation is as follows:

Forward (applicable to DxC 660i values ≤ 23 mg/L): Y (Cobas 6000) = 0.8695 (95% CI: 0.8419 to 0.8971) ∗ X (DxC 660i) + 0.2954 (95% CI: 0.2786 to 0.3121)

The NHANES performed complete blood cell counts (CBC) in duplicate for all study participants over one year of age. Blood samples were obtained by venipuncture into EDTA tubes and analyzed on a Coulter^®^DXH800 analyzer. Counts of WBC and their subtypes were obtained using a UNICEL DXH800 analyzer.

### 2.5. Sensitive Analysis

To make our results more representative, we performed a sensitivity analysis. BMI is the simplest and broadest anthropometric measure of general obesity [BMI = weight (kg)/height (m)^2^]. According to World Health Organization standards, general obesity is defined as BMI ≥ 30 kg/m^2^. Individuals with general obesity were included in the study as a sensitive analysis.

### 2.6. Covariates

Recent epidemiological studies have shown that dietary over-nutrition and institutionally driven declines in physical activity may be important factors influencing obesity [33,34]. Obesity is associated with an increased risk of diabetes, according to a meta-analysis involving 18 prospective studies [35]. Previous prospective studies have found that the development of high blood pressure is proportional to the level of obesity [36]. A cross-sectional study of 499,504 adults found that smoking was associated with an increased risk of obesity [37]. In addition, since dietary patterns and the state of obesity may vary by race and generation, we further adjusted for relevant demographic variables.

Trained NHANES investigators obtained demographic information from participants living in sample areas. To control for the effect of potential confounders, the following covariates were included: age (18–39, 40–59, >60), sex (men, women), race/ethnicity (Mexican American, Other Hispanic, non-Hispanic White, non-Hispanic Black, and Other Race), education of household referent (less than high school, high school, more than high school), ratio of family income to poverty, marital status (married/living with partner, widowed/divorced/separated/never married), work activity (vigorous activity, moderate activity, and other), recreational activity (vigorous activity, moderate activity, and other), smoking (never smoker, former smoker: lifetime intake of more than 100 cigarettes but current serum cotinine does not reach the threshold, current smoker: lifetime intake of more than 100 cigarettes and current serum cotinine reach the threshold), diabetes (self-reported whether they have ever been diagnosed with diabetes by a doctor), and hypertension (yes: systolic blood pressure ≥ 130 or diastolic blood pressure ≥ 80, or no). The threshold for serum cotinine, which is used to distinguish former smokers and current smokers, were for non-Hispanic white > 4.85 ng/mL, non-Hispanic Black > 5.92 ng/mL, Mexican American > 0.84 ng/mL and other > 3.08 ng/mL [38].

### 2.7. Statistical Analysis

The analysis was performed using Stata version 12.0 (Stata Corporation, College Station, TX, USA) and SAS version 9.4 (SAS Institute, Inc., Cary, NC, USA). Due to the complex sampling design, all analyses were adjusted for survey design and weight variables. Since this study combined NHANES data from 2 periods, a new sample weight (the original 2-year sample weight divided by 2) was constructed according to the NHANES analysis guidelines before analysis. Classified variables were described by percentage, and the basic characteristics of continuous variables were described by mean and standard deviation. Student’s *t*-test and rank sum test are used to analyze differences between continuous data, and chi-square test is used to analyze differences between classified data. The normality of each clinical biomarker was assessed based on visual inspection of the normogram and assessment of skewness and kurtosis measurements. If the results were not normal, they were naturally log-transformed. Residuals of the predicted values were plotted and assessed for normality. To better fit the model, both hs-CRP and NLR were naturally log-transformed.

**Figure 3:**
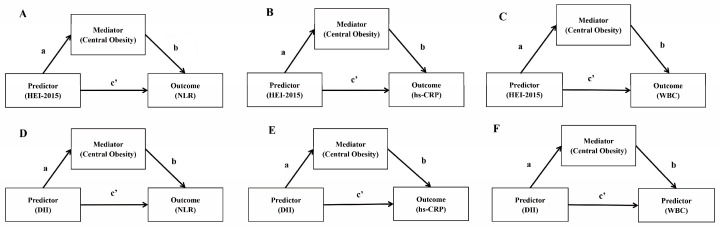
Mediation model path diagram. (**a**) The effect of dietary scores on central obesity; (**b**) the effect of central obesity on inflammatory markers; (**c**) direct effect of predictors on inflammatory markers. (**A**–**F**) show different potential paths, respectively.

All statistical analyses were based on the survey design and weighted variables adjusted to account for the complex sample design and to ensure nationally representative estimates. Multiple linear regression and multiple logistic regression analyses were used to explore the association between diet scores and obesity according to the type of study data. Weighted generalized structural equation models (GSEMs) were used to explore the mediating effect of central obesity on diet score and low-grade inflammation. We performed a sensitivity analysis on people with general obesity. The mediation model is constructed and analyzed by a causal diagram (Figure 3). All *p* values reported were two-sided; *p* < 0.05 was statistically significant.

## 3. Results

Table 1 shows the baseline characteristics of participants in terms of obesity and central obesity. A total of 8157 members were included in our study. The proportion of central obesity was 56.1%. Central obesity was found in 40.4% of men and 59.6 of women, and women were more likely to be more obese than men. There were statistically significant differences between people with central obesity or without central obesity in gender, age, race, education level, PIR, smoking, diabetes, work activities, recreational activities, and hypertension.

Table 2 shows the results of studies on the relationship between dietary score, general obesity, and central obesity on inflammatory markers. A multiple linear regression model was used to conduct statistical analysis on the results. The results in all adjusted models show that increased DII score was associated with the risk of hs-CRP and WBC (β_hs-CRP_ = 0.046, 95%CI: 0.025, 0.068; β_WBC_ = 0.058, 95%CI: 0.012, 0.103). Increased HEI score was associated with reduced risk of hs-CRP and WBC (β_hs-CRP_ = −0.006, 95%CI: −0.009, −0.004; β_WBC_ = −0.010, 95%CI: −0.015, −0.005). Increased risk of general obesity was associated with the increased level of hs-CRP and WBC (β_hs-CRP_ = 0.650, 95%CI: 0.591, 0.709; β_WBC_ = 0.502, 95%CI: 0.377, 0.627). The increased risk of central obesity was associated with the increased level of hs-CRP and WBC (β_hs-CRP_ = 0.661, 95%CI: 0.592, 0.730; β_WBC_ = 0.582, 95%CI: 0.440, 0.724). The association between NLR and both types of obesity and dietary score were not statistically significant (*p* > 0.05).

Table 3 shows the correlation between two different dietary scores and two types of obesity. Multiple logistic regression models were used to analyze the data. The results all adjusted model shows that increased HEI score was adversely associated with the risk of both types of obesity (OR_general obesity_ = 0.980, 95%CI: 0.975, 0.985; OR_central obesity_ = 0.987, 95% CI: 0.980, 0.994). Increased DII scores was associated with the risk of both two types of obesity (OR_general obesity_ = 1.066, 95%CI: 1.008, 1.127; OR_central obesity_ = 1.055, 95% CI: 1.002, 1.110). The association between changes in NLR and two types of obesity was not statistically significant (*p* > 0.05).

Table 4 shows the mediating effect analysis of central obesity on the relationship between dietary pattern and inflammatory markers in serum. The results showed that the mediating effect regression coefficient of central obesity on the relationship between DII score, HEI score and hs-CRP was statistically significant (β_DII_ = 0.007, 95%CI: 0.001, 0.014; β_HEI_ = −0.002, 95%CI: −0.003,−0.001), accounting for 15.24% and 26.87% of the total effect, respectively. The mediating effect of central obesity influence on the relationship between DII score, HEI score, and WBC was statistically significant (β_DII_ = 0.006, 95%CI: 0.000009, 0.012; β_HEI_ = −0.001, 95%CI: −0.002,−0.0005), accounting for 10.83% and 13.98% of the total effect, respectively.

Table 5 shows the mediating effect analysis of general obesity on the relationship between dietary pattern and inflammatory markers in serum, which was analyzed by using generalized structural equations. The sensitive analysis shows that the mediating effect still significant (*p* < 0.05).

## 4. Discussion

In this study, data from two periods of NHANES, 2015–2016 and 2017–2018, were used to analyze the mediating effect of central obesity on the relationship between dietary scores and low-grade inflammation-related serum inflammatory markers. We found that both DII and HEI-2015 dietary scores were associated with central obesity in US adults. In addition, central obesity partially mediated the association between dietary score and low-grade inflammation-related serum inflammatory markers. In the association of DII with hs-CRP and WBC, central obesity mediated 15.24% and 10.83%, respectively. Among the associations of HEI-2015 with hs-CRP and WBC, central obesity mediated 26.87% and 13.98%, respectively.

The effect of dietary quality and dietary inflammation index may be mediated partly by central obesity. We found that HEI-2015 score was negatively correlated with hs-CRP and WBC, and DII score was positively correlated with these two serum inflammatory markers. Hs-CRP and WBC were positively associated with obesity and central obesity, respectively. A prospective study found that dietary patterns are associated with pro-inflammatory and anti-inflammatory characteristics of gut microbial bacteria [39]. A British twin cohort showed that dietary quality was associated with methylation of 24 CpG sites, several of which were associated with adiposity, inflammation, and glucose abnormalities [40]. Alterations in HEI are associated with altered expression of genes that are markers of inflammation [41,42], and the effects of diet in regulating inflammation are thought to be due to complex interactions between food and biologically active nutrients [12]. A cross-sectional study of 20,823 adults at Moli-Sani constructed an INFLA composite score (CRP, white blood cell count, and NLR) that was positively associated with DII score [43].

Therefore, positive and effective diet for individuals with obesity may help to better maintain state of inflammation, and consequently avoid the occurrence and development of other complications. Dietary recommendations from the DGA can help people reduce their risk of obesity and low-grade inflammation. Several studies [44,45] have shown that a diet high in vegetables and fruits is inversely associated with inflammatory markers, while a diet high in meat, low in vegetables and omega-3 fatty acids, and high in refined carbohydrates, added sugars, saturated and trans fatty acids tends to be positively associated with inflammatory markers. The most obvious change from the 2015–2020 version of the DGA is the explicit limit on added sugars. Intake of added sugars, such as sucrose and high fructose corn syrup, has increased over the past hundred years and is strongly associated with increases in obesity, metabolic syndrome, and diabetes [46].

We found that HEI-2015 was significantly negatively associated with obesity. DII was significantly correlated with of obesity, and two different types of obesity were significantly correlated with hs-CRP and WBC levels. Obesity is a chronic, low-grade inflammatory state, and there may be several reasons why obesity leads to increased levels of inflammatory markers. Fat cells enhance insulin resistance and metabolic disorders, thereby promoting inflammation by increasing levels of CRP and other inflammatory markers [47]. Macrophages are reported to be the source of adipose tissue-derived proteins [48]. In individuals with abdominal obesity, an increase in the number of macrophages infiltrating visceral adipose tissue suggests that adipose tissue itself is a source and site of inflammation [49].

Based on this evidence, we found that dietary patterns and diet quality may influence low-level inflammation through obesity. Therefore, dietary intervention for obese individuals to improve their dietary quality can effectively avoid the risk of inflammation and prevent its complications. Of course, central obesity may not be the only mediator between diet quality and low-grade inflammation. Other factors, such as hypertension and diabetes, have also been strongly linked to diet quality and inflammation, and these need to be further verified in subsequent studies.

Our study has several advantages. First of all, the sample size we selected was large enough, and because the coverage of NHANES was very wide, the sample representation was very good. Second, we weighted the data throughout the study, helping to extrapolate our results to the entire U.S. population. Third, we conducted correlation analysis before mediation analysis to improve the credibility of the results.

At the same time, the disadvantages of the research should not be ignored. First of all, this is a cross-sectional study that cannot determine the causal relationship, and further prospective studies are needed to explore it. Second, the absence of data on the study population may lead to selection bias and affect the results of the whole study. Third, we use a limited set to work with in NHANES; thus, we could not further adjust for variables such as genetic factors or microbial factors that also have an impact. Finally, we used dietary review data and limited food groups to construct dietary scores, particularly DII, which may have influenced our results.

## 5. Conclusions

In conclusion, the results of this study suggest that better dietary quality can influence the state of central obesity, which can reduce the level of inflammation. It should be noted that dietary quality and dietary inflammatory factors may have important implications for the prevention of the level of inflammation, which should be further explored in prospective studies.

## Figures and Tables

**Table 1:** Characteristics of participants by central obesity in NHANES 2015–2018 (N = 8157).

	All Participants	Central Obesity		
		NON-Central Obesity	Central Obesity	*p* Value
Number of participants (%)	8157	3581	4576	
Age (%) ^a^				<0.001
18–39	2897 (35.5)	1692 (47.2)	1205 (26.3)	
40–59	2539 (31.1)	1026 (28.7)	1513 (33.1)	
>60	2721 (33.4)	863 (24.1)	1858 (40.6)	
Gender (%) ^a^				<0.001
Men	4122 (50.5)	2275 (63.5)	1847 (40.4)	
Women	4035 (49.5)	1306 (36.5)	2729 (59.6)	
Race/ethnicity (*n*, %) ^a^				<0.001
Mexican American	1312 (16.1)	461 (12.9)	851 (18.6)	
Other Hispanic	947 (11.6)	415 (11.6)	532 (11.6)	
Non-Hispanic White	2805 (34.4)	1078 (30.1)	1727 (37.7)	
Non-Hispanic Black	1724 (21.1)	726 (20.3)	998 (21.8)	
Other Race	1369 (16.8)	901 (25.2)	468 (10.2)	
Education Level (%) ^a^				<0.001
Less than high school	1578 (20.4)	643 (19.6)	935 (20.9)	
High school	1780 (23.0)	692 (21.1)	1088 (24.3)	
More than high school	4396 (56.7)	1940 (59.2)	2456 (54.8)	
Ratio of family income to poverty (PIR) (Mean ± SD) ^b^	2.54 ± 1.62	2.60 ± 1.66	2.49 ± 1.59	0.004
Marital status (%) ^a^				<0.001
Married	4004 (51.6)	2473 (51.9)	1531 (51.2)	
Living with partner	524 (6.8)	309 (6.5)	215 (7.2)	
Widowed	831 (10.7)	479 (10.1)	352 (11.8)	
Divorced	276 (3.6)	155 (3.3)	121 (4.0)	
Separated	1409 (18.2)	905 (19.0)	504 (16.9)	
Never married	712 (9.2)	444 (9.3)	268 (9.0)	
Diabetes (%) ^a^				<0.001
No	6875 (86.4)	3260 (92.7)	3615 (81.4)	
Yes	1080 (13.6)	256 (7.3)	824 (18.6)	
Work activities (%) ^a^				0.002
Vigorous activity	1961 (24.0)	905 (25.3)	1056 (23.1)	
Moderate activity	1813 (22.2)	737 (20.6)	1076 (23.5)	
Other	4383 (53.7)	1939 (54.1)	2444 (53.4)	
Recreational activities (%) ^a^				<0.001
Vigorous activity	2221 (27.2)	1356 (37.9)	865 (18.9)	
Moderate activity	1911 (23.4)	758 (21.2)	1153 (25.2)	
Other	3025 (49.3)	1467 (41.0)	2558 (55.9)	
Hypertension (%) ^a^				<0.001
No	4761 (59.4)	2363 (67.3)	2398 (53.2)	
Yes	3260 (40.6)	1149 (32.7)	2111 (46.8)	
Smoking (%) ^a^				<0.001
No smoker	4911 (60.2)	2228 (62.3)	2683 (58.7)	
Former smoker	1627 (20.0)	542 (15.1)	1085 (23.7)	
Current smoker	1614 (19.8)	809 (22.6)	805 (17.6)	
DII score ^c^	0.04 ± 1.60	−0.13 ± 1.64	0.17 ± 1.55	<0.001
HEI score ^b^	53.15 ± 13.93	53.54 ± 14.20	52.85 ± 13.71	0.026
hs-CRP ^c^	1.99 ± 0.97	1.57 ± 1.59	3.13 ± 2.28	<0.001
NLR ^c^	2.44 ± 2.15	1.94 ± 1.00	2.03 ± 0.95	<0.001
WEB ^b^	6.91 ± 1.69	6.59 ± 1.65	7.17 ± 1.67	<0.001

^a^ *p*-value was tested by chi-square test; ^b^ *p*-value was tested by student’s *t*-test; ^c^ *p*-value was tested by rank sum test.

**Table 2:** Partial regression coefficient (β) with 95 percent confidence intervals (CIs) for diet score, obesity, and inflammation markers.

	Crude ^a^			Model 1 ^a^			Model 2 ^a^		
	t	*p*-Value	β ^b^ (95%CI)	t	*p*-Value	β (95%CI)	t	*p*-Value	β (95%CI)
NLR ^c^									
DII score	1.11	0.276	0.005 (−0.004, 0.014)	1.55	0.068	0.008 (−0.001, 0.016)	1.35	0.187	0.007 (−0.003, 0.016)
HEI score	−1.56	0.129	−0.001 (−0.002, 0.0002)	−3.35	0.002	−0.002 (−0.003, −0.001)	−1.53	0.138	−0.001 (−0.002, 0.0003)
General obesity	3.70	0.001	0.044 (0.020, 0.069)	2.99	0.005	0.034 (0.011, 0.057)	1.98	0.057	0.027 (−0.001, 0.054)
Central obesity	5.07	<0.001	0.065 (0.039, 0.091)	2.92	0.007	0.036 (0.011, 0.062)	1.16	0.255	0.016 (−0.012, 0.044)
Hs-CRP ^d^									
DII score	9.61	<0.001	0.076 (0.060, 0.092)	8.51	<0.001	0.070 (0.053, 0.087)	4.43	<0.001	0.046 (0.025, 0.068)
HEI score	−7.57	<0.001	−0.007 (−0.009, −0.005)	−10.50	<0.001	−0.009 (−0.011, −0.007)	−5.20	<0.001	−0.006 (−0.009, −0.004)
General obesity	26.09	<0.001	0.741 (0.683, 0.799)	26.65	<0.001	0.732 (0.676, 0.788)	22.56	<0.001	0.650 (0.591, 0.709)
Central obesity	25.22	<0.001	0.756 (0.695, 0.818)	24.29	<0.001	0.740 (0.678, 0.803)	19.56	<0.001	0.661 (0.592, 0.730)
WBC ^e^									
DII score	5.09	<0.001	0.096 (0.057, 0.134)	4.66	<0.001	0.095 (0.053, 0.136)	2.56	0.016	0.058 (0.012, 0.103)
HEI score	−7.34	<0.001	−0.016 (−0.020, −0.012)	−7.27	<0.001	−0.016 (−0.021, −0.012)	−4.01	<0.001	−0.010 (−0.015, −0.005)
General obesity	11.30	<0.001	0.618 (0.507, 0.730)	11.73	<0.001	0.632 (0.522, 0.742)	8.19	<0.001	0.502 (0.377, 0.627)
Central obesity	10.68	<0.001	0.626 (0.506, 0.746)	12.62	<0.001	0.712 (0.597, 0.828)	8.37	<0.001	0.582 (0.440, 0.724)

^a^ Calculated using liner regression. Model 1 adjusted for age and gender. Model 2 adjusted for age and gender, race/ethnicity, education, marital status, poverty ratio, diabetes, work activities, recreational activities, hypertension, and smoking. ^b^ β: partial regression coefficient. ^c^ NLR: neutrophils/lymphocytes after logarithmic conversion; ^d^ hs-CRP: high-sensitivity c-reactive protein after logarithmic conversion; ^e^ WBC: white blood cells.

**Table 3:** Weighted odds ratios (ORs) with 95 percent confidence intervals (CIs) for dietary quality, dietary inflammation level and obesity.

	Crude ^a^			Model 1 ^a^			Model 2 ^a^		
	t	*p*-Value	β ^b^ (95%CI)	t	*p*-Value	β (95%CI)	t	*p*-Value	β (95%CI)
General obesity									
DII score	3.41	0.002	1.088 (1.034, 1.144)	3.91	<0.001	1.100 (1.047, 1.157)	2.35	0.026	1.066 (1.008, 1.127)
HEI score	−10.74	<0.001	0.980 (0.976, 0.984)	−11.24	<0.001	0.977 (0.973, 0.981)	−7.92	<0.001	0.980 (0.975, 0.985)
Central obesity									
DII score	4.55	<0.001	1.121 (1.065, 1.180)	3.33	0.002	1.086 (1.032, 1.142)	2.12	0.042	1.055 (1.002, 1.110)
HEI score	−2.75	0.010	0.993 (0.988, 0.998)	−6.86	<0.001	0.982 (0.976, 0.987)	−3.60	0.001	0.987 (0.980, 0.994)

^a^ Calculated using logistic regression. Model 1 adjusted for age and gender. Model 2 adjusted for age and gender, race/ethnicity, education, marital status, poverty ratio, diabetes, work activities, recreational activities, hypertension, and smoking. ^b^ OR: odd ratio.

**Table 4:** The weighted mediating central obesity on the association between dietary score and inflammation markers.

	Indirect Effect (95%CI)	Total Effect (95%CI)	Proportion Mediated (%)
DII score			
NLR ^a^	−0.0002 (−0.0002, 0.0005)	0.007 (−0.003, 0.016)	2.53
hs-CRP ^b^	0.007 (0.001, 0.014) *	0.046 (0.026, 0.067) ***	15.24
WBC ^c^	0.006 (0.000009, 0.012) *	0.058 (0.014, 0.102) *	10.83
HEI score			
NLR	−0.0004 (−0.0001, 0.00004)	−0.0009 (−0.002, 0.0003)	3.95
hs-CRP	−0.002 (−0.003, −0.001) ***	−0.006 (−0.009, −0.004) ***	26.87
WBC	−0.001 (−0.002, −0.0005) **	−0.010 (−0.015, −0.005) ***	13.98

Adjusted for age and gender, race/ethnicity, education, marital status, poverty ratio, diabetes, work activities, recreational activities, hypertension, and smoking. ^a^ NLR: neutrophils/lymphocytes after logarithmic conversion; ^b^ hs-CRP: high-sensitivity c-reactive protein after logarithmic conversion; ^c^ WBC: white blood cells. * *p* < 0.05, ** *p* < 0.01, *** *p* < 0.001.

**Table 5:** The weighted mediating general obesity on the association between dietary score and inflammation markers.

	Indirect Effect (95%CI)	Total Effect (95%CI)	Proportion Mediated (%)
DII score			
NLR ^a^	0.0004 (−0.0001, 0.0008)	0.007 (−0.003, 0.016)	5.44
hs-CRP ^b^	0.009 (0.002, 0.016) *	0.046 (0.026, 0.067) ***	19.13
WBC ^c^	0.058 (0.014, 0.102) *	0.007 (0.001, 0.013) *	11.86
HEI score			
NLR	−0.0001 (−0.0002, 0.00002)	−0.001 (−0.002, 0.0003)	11.00
hs-CRP	−0.0027 (−0.003, −0.002) ***	−0.006 (−0.009, −0.004) ***	43.49
WBC	−0.002 (−0.003, −0.001) ***	−0.010 (−0.015, −0.005) ***	19.57

Adjusted for age and gender, race/ethnicity, education, marital status, poverty ratio, diabetes, work activities, recreational activities, hypertension, and smoking. ^a^ NLR: neutrophils/lymphocytes after logarithmic conversion; ^b^ hs-CRP: high-sensitivity c-reactive protein after logarithmic conversion; ^c^ WBC: white blood cells. * *p* < 0.05, *** *p* < 0.001.

## Data Availability

The data are available at https://www.cdc.gov/nchs/nhanes/index.htm (accessed on 11 December 2022).
